# An improved method for rapid detection and characterization of carbapenemase-producing Enterobacterales directly from positive blood cultures: dBLIMplus

**DOI:** 10.1128/spectrum.01913-25

**Published:** 2026-04-21

**Authors:** Tugba Ayvalik Ruzgarkesen, Emel Sesli Cetin, Mumtaz Cem Sirin, Buket Aridogan

**Affiliations:** 1 Medical Microbiology, Cizre Dr. Selahattin Cizrelioglu State Hospital 652430 , Cizre, Şırnak, Turkey; 2 Department of Medical Microbiology, Suleyman Demirel University Faculty of Medicine, Isparta, Turkey; bioMerieux Inc, Eugene, Oregon, USA

**Keywords:** blood culture, carbapenemases, combination disks, phenotypic method, rapid detection

## Abstract

In this study, a new phenotypic method “dBLIMplus” performed by using combination disks for both detection of carbapenemase activity and determination of the carbapenemase enzyme of carbapenemase-producing Enterobacterales strains from positive blood culture bottles was introduced and evaluated relative to the carbapenemase enzyme determined by the BD MAX Check-Points CPO test. Sixty strains (*bla*_KPC_ [*n* = 15], *bla*_NDM_ [*n* = 13], *bla*_VIM/IMP_ [*n* = 2], *bla*_OXA-48_ [*n* = 15], and non-carbapenemase-producing Enterobacterales [*n* = 15]) were used for validation of dBLIMplus, and zone diameter interpretation criteria were determined. dBLIMplus was applied to 100 Enterobacterales clinical isolates to evaluate its applicability in routine laboratory practices. The bacterial suspension to be tested was obtained by stepwise centrifugation applied to the blood culture fluid. Two hundred microliters of the suspension was added to D73C-MASTDISCS Combi Carba Plus disks. Simultaneously, 200 microliters of *Escherichia coli* ATCC 25922 reference strain adjusted to 1.0 McFarland was transferred to Mueller-Hinton agar, and all were incubated at 35°C ± 2°C for 2 h. After incubation, disks were placed on preincubated Mueller-Hinton agar and then incubated at 35°C ± 2°C. Inhibition zone diameters were measured at 6, 12, and 18 h and interpreted according to the evaluation criteria determined by validation. The sensitivity and specificity of dBLIMplus for detecting carbapenemase-producing strains and for identifying the individual carbapenemase enzymes were as follows: 100% and 100% for carbapenemase producers; 77.8% and 100% for *bla*_KPC_; 22.6% and 100% for metallo-beta-lactamases; and 79.2% and 98.1% for *bla*_OXA-48_. In conclusion, although the sensitivity of dBLIMplus is relatively low for isolates harboring more than one carbapenemase enzyme, it may still serve as a useful phenotypic tool in laboratories where PCR is not available thanks to its 100% sensitivity in detecting carbapenemase activity, nearly 80% sensitivity, and close to 100% specificity in identifying *bla*_KPC_ and *bla*_OXA-48_.

**IMPORTANCE:**

The significance of this study lies in the development and validation of a novel phenotypic method, dBLIMplus, for the rapid detection of carbapenemase-producing Enterobacterales directly from positive blood culture bottles. Carbapenemase-producing Enterobacterales are associated with high morbidity and mortality rates. Rapid and accurate identification is critical for timely infection control and effective treatment decisions. Given its ability to detect carbapenemase producers directly from positive blood culture bottles using only combination disks, dBLIMplus is presented as a viable alternative to molecular techniques, which are generally more costly. With 100% sensitivity and specificity in detecting carbapenemase-producing Enterobacterales, dBLIMplus demonstrates strong potential for routine clinical use. Its ability to deliver accurate results as early as 6 h makes it a valuable tool for microbiology laboratories, especially in resource-limited settings.

## INTRODUCTION

Antimicrobial resistance is reported by the World Health Organization (WHO) as one of the most important problems threatening public health (1, 2). Resistance is also increasing against carbapenems that are last-resort drugs in the treatment of infections caused by Enterobacterales (3–5). In the “Bacterial Priority Pathogens List” published in 2024 by the WHO, carbapenem-resistant *Klebsiella* spp. and *Escherichia coli* isolates were included in the critical-priority group. Early detection and characterization of carbapenemase enzyme production, the most common mechanism of carbapenem resistance in Enterobacterales, is important both for optimal treatment and infection control of the disease and for preventing the spread of resistance (6–9).

The European Committee on Antimicrobial Susceptibility Testing (EUCAST) recommends the use of phenotypic methods to detect carbapenemase production in Enterobacterales isolates, when decreased susceptibility to carbapenems is detected in routine susceptibility tests (10). Combination disk test (CDT), carbapenem inactivation method (CIM), biochemical (colorimetric) tests, immunochromatographic lateral flow tests, and Matrix-Assisted Laser Desorption/Ionization Time-of-Flight Mass Spectrophotometry (MALDI-TOF MS) are among the recommended phenotypic tests. Molecular tests used to detect carbapenemase production are considered the gold standard. However, they are expensive; moreover, they target only specific carbapenemase genes, and experienced personnel are required to perform these tests (11).

Although the CDT method has been validated in previous studies, provides information on the carbapenemase enzyme type, and can be performed using commercially available disks, it has certain limitations. One major limitation is that the results require up to 18 h to be read. This prolonged turnaround time has prompted researchers to develop faster tests for routine clinical application. Van der Zwaluw et al. introduced CIM as a sensitive, specific, and inexpensive test for the detection of carbapenemase-producing Enterobacterales (CPE), and Clinical and Laboratory Standards Institute (CLSI) subsequently approved CIM for the detection of carbapenemase production in 2015 with some modifications (12, 13). Many studies have been conducted to modify CIM in order to increase the sensitivity and specificity of the test, and CLSI approved the modified carbapenem inactivation method (mCIM) in 2017 and ethylene diamine tetraacetic acid (EDTA) carbapenem inactivation method (eCIM) in 2018 (14–18). mCIM is used to detect carbapenemase activity, while eCIM is used in conjunction with mCIM to distinguish metallo-β-lactamases (MBLs) from serine carbapenemases. Additional studies have reported poor sensitivity with eCIM in isolates containing both serine carbapenemases and MBLs. Moreover, a limitation of CIM and similar methods is that they require the use of isolated bacterial colonies in culture (6).

Further studies have been performed to evaluate the methods for rapid detection of carbapenemase activity. In 2020, Bianco et al. designed a new method inspired by CIM for the rapid detection of carbapenemase or extended-spectrum beta-lactamase (ESBL) activity directly from positive blood culture (BC) bottles in Enterobacterales isolates, and experimental evidence was presented confirming that CPE could be detected from positive BC bottles in less than 7 h with the method called “the direct beta-lactam inactivation method” (dBLIM). This was the first study reporting that CPE isolates could be directly detected from positive BC bottles. The dBLIM demonstrated over 99% sensitivity in detecting carbapenemase activity associated with *bla*_KPC_, *bla*_OXA-48_, and *bla*_NDM_ genes in both aerobic and anaerobic bottles. For *bla*_VIM_, the assay exhibited 100% sensitivity in aerobic bottles but only 53.6% sensitivity in anaerobic bottles. Failure to determine carbapenemase type by dBLIM and, in particular, low sensitivity for *bla*_VIM_ under anaerobic conditions was reported as limitations of the study (19).

The present study was based on the hypothesis that dBLIM could be used for determining the carbapenemase enzyme through the use of combination disks. The aim of the study was to evaluate the efficacy of a modified method, named dBLIMplus, which integrates the dBLIM with phenotypic combination disk testing to enable the rapid detection of CPE strains directly from positive BC bottles and to determine their carbapenemase enzyme types. This approach is expected to support antimicrobial management and strengthen infection control practices.

## MATERIALS AND METHODS

This study was approved by the Ethics Committee of the Faculty of Medicine, Suleyman Demirel University (Approval date: 16 February 2023; Decision No: 3/28).

### Selection and identification of bacterial strains

In this study, Enterobacterales strains obtained from various clinical samples (blood, urine, sputum, endotracheal aspirate, wound, and abscess) in the Bacteriology Laboratory of the Department of Medical Microbiology, Süleyman Demirel University Faculty of Medicine, between January 2022 and January 2024 were used. Identification and antimicrobial susceptibilities of all strains were performed by using the BD Phoenix 100 (Becton, Dickinson and Company, USA) automated system. To detect carbapenemase production phenotypically, mCIM was performed on all strains according to CLSI recommendations (20). To determine the carbapenemase enzyme phenotypically, CDT was performed by using the D73C-MASTDISCS Combi Carba Plus Disk Set (Mast Diagnostics, Liverpool, United Kingdom) according to the manufacturer’s recommendations. Additionally, to determine the carbapenemase enzyme genotypically, polymerase chain reaction (PCR) was performed using the BD MAX Check-Points CPO test (Becton, Dickinson and Company, USA). Considering the Power analysis data, a total of 60 Enterobacterales strains were included in the study as validation strains. dBLIMplus was applied to the validation strains, and dBLIMplus evaluation zone diameter criteria were determined. After validation was completed, 100 Enterobacterales clinical strains were selected to evaluate the applicability of dBLIMplus in routine laboratory practice, and dBLIMplus was applied to clinical strains. Only one bacterial strain isolated from each patient was included in the study.

### Transfer of bacteria into blood culture bottles

Aerobic BC bottles sent to the laboratory from various clinics and remained as negative after 5 days of incubation were removed from the Render BC128 (Shandong Huifa Electronics Technology, China) automated BC system. Bacterial isolates from frozen stocks were cultured overnight on 5% sheep blood agar at 35°C ± 2°C. Subsequently, 0.5 mL of a bacterial suspension containing 10³ CFU/mL was inoculated into a negative BC bottle and incubated in the same automated system. Positive BCs were subjected to dBLIMplus and subcultured onto 5% sheep blood agar to confirm the growth of the tested bacterial strains and to verify their purity.

### dBLIMplus application

**Fig 1:**
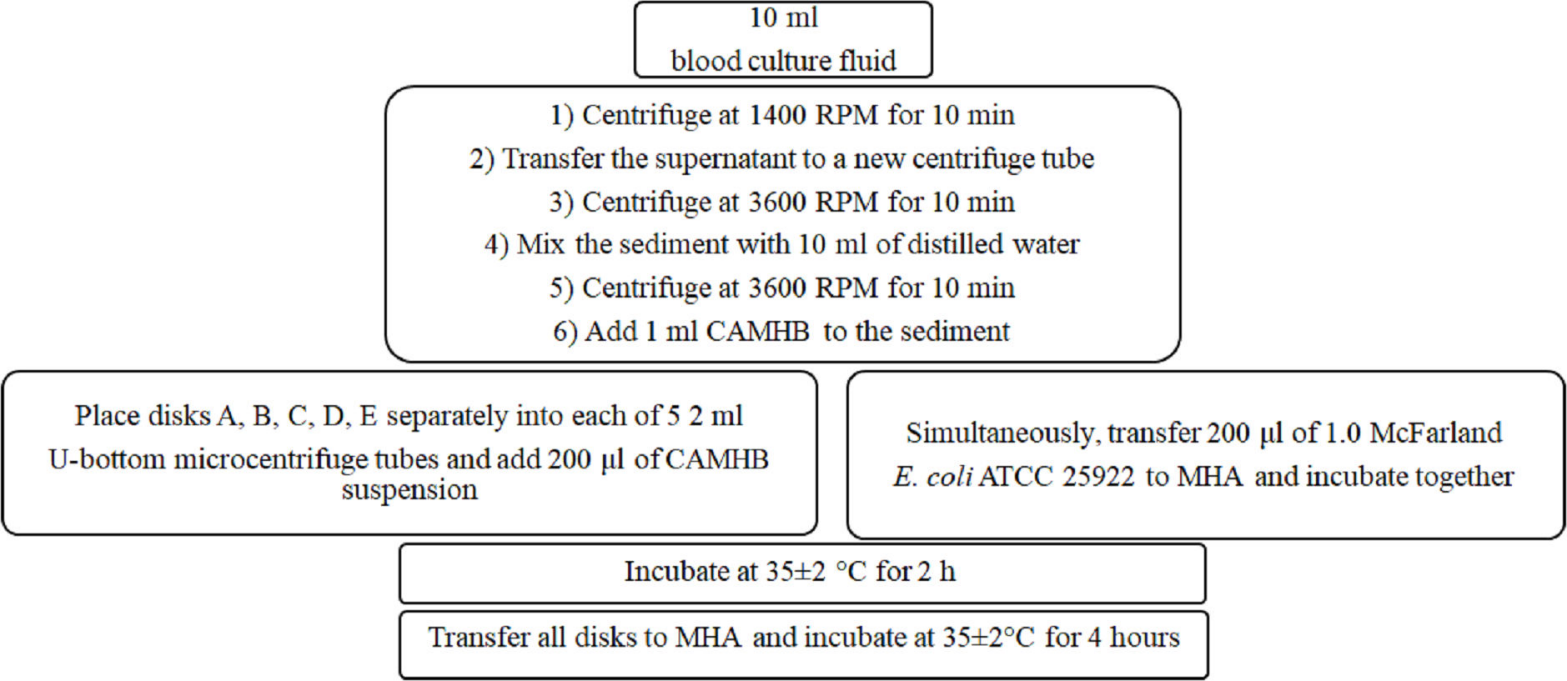
Workflow of the dBLIMplus method.

The working steps of dBLIMplus applied to positive BCs are summarized in Fig. 1. In detail, 10 mL of the culture fluid was withdrawn from the positive BC bottle, transferred to a sterile 15 mL centrifuge tube, and then centrifuged at 1,400 rpm for 10 min. The supernatant was transferred to a 15 mL sterile centrifuge tube and centrifuged at 3,600 rpm for 10 min. The supernatant was discarded with a sterile Pasteur pipette, and the sediment was suspended with 10 mL distilled water and centrifuged again for 10 min at 3,600 rpm. The supernatant was discarded, and the sediment was suspended with 1 mL extraction buffer (cation-adjusted Mueller-Hinton broth [CAMHB]). The bacterial suspension to be tested was obtained by this stepwise centrifugation method applied to the positive BC fluid. D73C-MASTDISCS Combi Carba Plus antibiotic disks (A, B, C, D, and E) were placed in 5 sterile 2 mL U-bottom microcentrifuge tubes, and 200 uL of the obtained suspension was added to them. Then, the tubes were incubated at 35°C ± 2°C for 2 h. Simultaneously, 200 uL of *E. coli* ATCC 25922 reference strain adjusted to 1.0 McFarland standard was transferred to the Mueller-Hinton agar (MHA) plate, spread equally on all sides of the plate, and incubated in parallel with the antibiotic combination disks under aerobic atmospheric conditions at 35°C ± 2°C for 2 h. After incubation, the disks were removed with a 10 uL disposable loop by dragging the ring along the inner edge of the microcentrifuge tube to expel excess liquid from the disk and placed on preincubated MHA plates, which were then incubated at 35°C ± 2°C. During incubation, inhibition zone diameters were measured visually from the front of the plate under reflected light by removing the plate lid and noted at 6, 12, and 18 h. To evaluate objectively, all visual evaluations were performed by two different researchers.

### Validation of dBLIMplus

A total of 60 Enterobacterales strains (*K. pneumoniae* [*n* = 49] and *E. coli* [*n* = 11]) consisting of 45 CPE and 15 non-carbapenemase-producing Enterobacterales (non-CPE) strains were used in the dBLIMplus validation. CPE isolates were selected based on resistance to all three carbapenems (meropenem [MEM], ertapenem, and imipenem), positivity in mCIM, and concordance between the carbapenemase enzyme identified by CDT and the resistance gene detected by PCR, while non-CPE isolates were selected from strains susceptible to all three carbapenems, negative in mCIM, and negative for carbapenemase production in both CDT and PCR. The 60 validation strains were categorized into four groups based on PCR results: 15 *bla*_KPC_, 15 MBL (13 *bla*_NDM_ and 2 *bla*_VIM/IMP_), 15 *bla*_OXA-48_, and 15 non-CPE (*bla*_KPC_, *bla*_OXA-48_, *bla*_NDM,_ and *bla*_VIM/IMP_-negative). To evaluate intertest and intratest variability, one strain from each group was selected, and dBLIMplus testing was independently performed and evaluated by two researchers. No significant differences were observed between the measured zone diameters. Subsequently, dBLIMplus was applied individually to all 60 validation strains, and inhibition zone diameters and zone diameter differences for each disk were recorded at 6, 12, and 18 h. The evaluation criteria for dBLIMplus were established based on the zone diameter ranges and zone diameter differences obtained for each disk within the validation groups.

### dBLIMplus assessment of clinical strains

After validation, a total of 100 clinical Enterobacterales strains, including both CPE and non-CPE isolates, were selected to assess the applicability of dBLIMplus in routine laboratory practice. CPE isolates were selected based on resistance to all three carbapenems, positivity in mCIM, and the presence of carbapenemase genes detected by PCR, while non-CPE isolates were selected from strains susceptible to all three carbapenems, negative in mCIM, and without carbapenemase genes by PCR. CDT and dBLIMplus were applied simultaneously on all these strains.

### CDT application with D73C-MASTDISCS Combi Carba Plus disk set

The antibiotics included in the D73C-MASTDISCS Combi Carba Plus carbapenemase disk set were as follows: A: penem 10 µg, B: penem 10 µg + MBL inhibitor, C: penem 10 µg + KPC inhibitor, D: penem 10 µg + AmpC inhibitor, E: temocillin + MBL inhibitor. The tests were performed according to the manufacturer’s recommendations. Inhibition zone diameter differences were assessed both manually and using the D73C-Carba_Plus_Calculator_V2.4.xlsx, an Excel-based program obtained from the MAST Group website (https://www.mast-group.com/).

### Statistical analysis

The power analysis of the study was performed by the GPower 3.1.9.6 program (Universitaet Kiel, Germany). The data obtained in the study were analyzed by using the SPSS 26.0 program. A *P* value of < 0.05 was considered statistically significant. The sensitivity of dBLIMplus was determined by accepting PCR as the gold-standard method. McNemar test was used to determine whether there was a significant difference among the 6-, 12-, and 18-h readings of dBLIMplus. Additionally, Cohen’s kappa analysis was used to determine the agreement of dBLIMplus and CDT results with PCR.

## RESULTS

### dBLIMplus validation

mCIM, CDT, and dBLIMplus findings of validation strains grouped according to the carbapenemase type determined by PCR are presented in Table 1. The dBLIMplus test was applied separately to each validation strain, and the inhibition zone diameters and differences in zone diameters of all disks were recorded at 6, 12, and 18 h (Table S1). When the four groups (non-CPE, *bla*_KPC,_ MBL, and *bla*_OXA-48_) were evaluated separately in terms of dBLIMplus results, although there were small differences in terms of zone diameters and zone diameter differences between the results obtained at 6, 12, and 18 h, no statistically significant difference was observed across the three reading periods (*P* > 0.05). Evaluation criteria for dBLIMplus were determined based on zone diameters and zone diameter differences of each disk calculated at 6, 12, and 18 h for four groups. Carbapenemase activity was interpreted based on the zone diameter of disk A (Z_A_). While Z_A_≤11 mm was considered indicative of carbapenemase production, Z_A_ > 11 mm was interpreted as no carbapenemase activity. When Z_A_ was detected as ≤11 mm, the differences in zone diameters between the disks were evaluated to identify the carbapenemase type. A strain was classified as MBL-producing when Z_B-A_ was ≥5 mm, and both disks Z_C-A_ and Z_D-A_ were <5 mm. A strain was classified as KPC-producing when Z_C-A_ was ≥5 mm, and both disks Z_B-A_ and Z_D-A_ were <5 mm. A strain was classified as OXA-48-producing when Z_B-A_, Z_C-A_, and Z_D-A_ were each <5 mm, and Z_E_ was ≤11 mm. Results that could not be classified in any group were defined as “indeterminate.” The evaluation criteria are shown as a diagram in Fig. S1.

**TABLE 1:** mCIM, CDT, and dBLIMplus findings of validation strains grouped according to the carbapenemase type determined by PCR

Carbapenemase type	mCIM	CDT	dBLIMplus (6–12–18 h)
Non-CPE (*n* = 15)	Negative (*n* = 15)	Non-CPE (*n* = 15)	Non-CPE (*n* = 15)
*bla*_KPC_ (*n* = 15)	Positive (*n* = 15)	KPC (*n* = 15)	KPC (*n* = 15)
MBL (*n* = 15)	Positive (*n* = 15)	MBL (*n* = 15)	MBL (*n* = 15)
*bla*_OXA-48_ (*n* = 15)	Positive (*n* = 15)	OXA-48 (*n* = 15)	OXA-48 (*n* = 15)

### dBLIMplus assessment of clinical strains

Of the 100 clinical strains (66 *K. pneumoniae* and 34 *E. coli*) included in the study, 56 were detected as CPE and 44 as non-CPE by PCR. Clinical strains were divided into five groups (*bla*_NDM+OXA-48_ [*n* = 31], *bla*_OXA-48_ [*n* = 16], *bla*_KPC_ [*n* = 8], *bla*_KPC+OXA-48_ [*n* = 1], and non-CPE [*n* = 44]) according to the carbapenemase type. The ranges of zone diameters and zone diameter differences measured with dBLIMplus for each group at 6, 12, and 18 h are presented in Table S2. The zone diameters of the disk A for clinical strains ranged from 12 to 14 mm in the non-CPE group and from 6 to 11 mm in the CPE group. Both the sensitivity and specificity of dBLIMplus in detecting CPE were determined as 100%. While 7 of 8 *bla*_KPC_ strains were correctly identified as KPC-producing by dBLIMplus, one of them was identified as OXA-48-producing at all time points, with a Z_C-A_ measurement of 3 mm. On the other hand, 12 of 16 *bla*_OXA-48_ strains were identified as OXA-48-producing by dBLIMplus at all time points, while one strain showed a Z_E_ of 12 mm at all hours, and the dBLIMplus result was interpreted as indeterminate. Three strains had a Z_E_ of 10 mm at the 6-h time point and were considered OXA-48-producing by dBLIMplus; however, at the 12- and 18-h time points, the Z_E_ increased to 12 mm, and the results were interpreted as indeterminate. One *bla*_KPC+OXA-48_ strain was identified as OXA-48-producing by dBLIMplus. Among the 31 *bla*_NDM+OXA-48_ strains, 22 were identified as OXA-48-producing and seven as MBL-producing by dBLIMplus. In two strains, Z_B-A_ and Z_D-A_ were ≥5 mm at all time points; therefore, these strains were interpreted as indeterminate by dBLIMplus. The mCIM, CDT, and dBLIMplus findings of clinical strains classified according to carbapenemase resistance genes determined by PCR are given in Table 2. dBLIMplus was applied a second time to the strains that gave indeterminate results with dBLIMplus, and the same results were obtained. The sensitivity, specificity, positive predictive value (PPV), and negative predictive value (NPV) of dBLIMplus in detecting the carbapenemase type/types of the tested strains were 39.3%, 100%, 100%, and 56.4% at 6 h and 33.9%, 100%, 100%, and 54.3% at 12 and 18 h, respectively. With dBLIMplus, all carbapenemase types of 56 carbapenemase-producing isolates could be detected completely in 22 isolates (39.3%) at the 6-h and in 19 isolates (33.9%) at the 12- and 18-h time points. dBLIMplus showed 77.8% sensitivity and 100% specificity for *bla*_KPC_, 22.6% sensitivity and 100% specificity for MBL, and 79.2% sensitivity and 98.1% specificity for *bla*_OXA-48_ carbapenemase. No statistically significant differences were observed in dBLIMplus results between all hour readings for *bla*_KPC_, MBL, and *bla*_OXA-48_ isolates (*P* > 0.05). The agreement between dBLIMplus and PCR results for all isolates was assessed using Cohen’s kappa analysis. When the agreement between the dBLIMplus and PCR results was evaluated, a weak-to-moderate level of concordance was observed between the two methods (ĸ = 0.363; *P* < 0.001). dBLIMplus was unable to fully identify all carbapenemase types in 32 CPE isolates harboring more than one carbapenemase gene. Of the 24 CPE isolates harboring a single carbapenemase gene, dBLIMplus correctly identified the carbapenemase type in 22 isolates (91.6%) at 6 h and in 19 isolates (79.2%) at 12 and 18 h. The sensitivity and specificity of dBLIMplus for detecting the carbapenemase enzyme type in strains harboring a single carbapenemase gene were 87.5% and 100% for *bla*_KPC_ at all hours; 93.5% and 100% for *bla*_OXA-48_ at the 6-h time point; and 75% and 100% at the 12- and 18-h time points, respectively. Since none of the clinical strains contained *bla*_NDM_ alone, an evaluation of *bla*_NDM_ could not be performed.

**TABLE 2:** mCIM, CDT, and dBLIMplus findings of clinical strains classified according to carbapenemase resistance genes determined by PCR

Carbapenemase type	mCIM	CDT	dBLIMplus (6 h)	dBLIMplus (12–18 h)
*bla*_KPC_ (*n* = 8)	Positive (*n* = 8)	KPC (*n* = 4) Indeterminate (*n* = 3) OXA-48 (*n* = 1)	KPC (*n* = 7) OXA-48 (*n* = 1)	KPC (*n* = 7) OXA-48 (*n* = 1)
*bla*_KPC+OXA-48_ (*n* = 1)	Positive (*n* = 1)	KPC (*n* = 1)	OXA-48 (*n* = 1)	OXA-48 (*n* = 1)
*bla*_NDM+OXA-48_ (*n* = 31)	Positive (*n* = 31)	MBL (*n* = 18) OXA-48 (*n* = 13)	OXA-48 (*n* = 22) MBL (*n* = 7) Indeterminate (*n* = 2)	OXA-48 (*n* = 22) MBL (*n* = 7) Indeterminate (*n* = 2)
*bla*_OXA-48_ (*n* = 16)	Positive (*n* = 16)	OXA-48 (*n* = 15) MBL (*n* = 1)	OXA-48 (*n* = 15) Indeterminate (*n* = 1)	OXA-48 (*n* = 12) Indeterminate (*n* = 4)
non-CPE (*n*=44)	Negative (*n* = 44)	non-CPE (*n* = 44)	non-CPE (*n* = 44)	non-CPE (*n* = 44)

Examples of dBLIMplus inhibition zone diameters of non-CPE (Fig. S2A through C), OXA-48-positive (Fig. S2D through F), MBL-positive (Fig. S2G through I) and KPC-positive (Fig. S2J through L) strains at 6, 12, and 18 h are shown in Fig. S2, respectively.

### CDT results of clinical strains

Carbapenemase activity was detected by CDT in 53 (94.6%) of the 56 strains identified as CPE by PCR. Carbapenemase activity was detected by CDT in 21 (87.5%) of 24 CPE isolates harboring a single carbapenemase type, and the carbapenemase type was correctly identified by CDT in 19 (79.1%) of them. Among the *bla*_KPC_ isolates, 4 of 8 clinical strains showed complete concordance with CDT results, one strain was identified as OXA-48-producing by CDT, and three strains were interpreted as indeterminate. For these three indeterminate strains, the Z_C-A_ measurements were 2 mm, 2 mm, and 3 mm, respectively; however, these values were insufficient to identify them as KPC-producing isolates. The isolate identified as OXA-48-producing by CDT was also detected as OXA-48-producing by dBLIMplus at all time points. One *bla*_KPC+OXA-48_ isolate was detected as KPC-producing by CDT and OXA-48-producing by dBLIMplus. Of the 31 *bla*_NDM+OXA-48_ isolates, 18 were detected as MBL-producing and 13 as OXA-48-producing by CDT. Of the isolates tested, 11 that were identified as MBL-producing by CDT were detected as OXA-48-producing by dBLIMplus across all reading time points, while two isolates identified as OXA-48-producing by CDT yielded indeterminate results with dBLIMplus at all time points. Fifteen of the 16 *bla*_OXA-48_ isolates showed full compatibility with CDT, while one was identified as MBL-producing. This isolate was detected as OXA-48-producing by dBLIMplus. The sensitivity, specificity, PPV, and NPV of CDT in determining the carbapenemase enzyme type were found as 33.9%, 100%, 100%, and 54.3%, respectively. CDT was determined to have 55.5% sensitivity and 100% specificity in detecting *bla*_KPC_ carbapenemases, 58.1% sensitivity and 98.5% specificity in detecting MBL carbapenemases, and 58.3% sensitivity and 98.1% specificity in detecting *bla*_OXA-48_ carbapenemases. The carbapenemase types determined by CDT and PCR were compared, and a statistically significant difference was found between the findings of the two methods (McNemar; *P* = 0.025). When the agreement between the CDT and PCR results was evaluated, a statistically significant but low-to-moderate level of concordance was observed between the two methods (ĸ = 0.291; *P* < 0.001). Carbapenemase activity was detected by CDT in 21 (87.5%) of 24 CPE isolates harboring a single carbapenemase type, and the carbapenemase type was correctly identified by CDT in 19 (79.1%) of them. The sensitivity and specificity of CDT for detecting the carbapenemase type in strains harboring a single carbapenemase gene were 50% and 100% for *bla*_KPC_ and 93.75% and 98.8% for *bla*_OXA-48_, respectively.

## DISCUSSION

Early detection of CPE is critical for infection control and prevention (21). Treatment options vary according to the carbapenemase enzyme type produced by CPE isolates; therefore, rapid determination of the carbapenemase enzyme type provides an important contribution to the management of the treatment. In this study, “dBLIMplus,” which is based on the use of a combination disk set to directly detect CPE strains from positive BC bottles and to determine the type of carbapenemase enzyme produced, was introduced as a new phenotypic method, and the effectiveness of the method was validated and evaluated by testing with strains whose carbapenemase enzymes were determined by PCR. This method originates from dBLIM, described by Bianco et al. to detect CPE isolates directly from positive BC bottles within 6 h. The reason why this method is called dBLIMplus is that the determination of carbapenemase enzyme (KPC, MBL, and OXA-48) is done by adding combination disks to dBLIM. Aerobic BC bottles were preferred for dBLIMplus based on the findings that aerobic BC bottles provide better results than anaerobic bottles in the detection of carbapenemase activity by dBLIM (19). Bianco et al. reported that dBLIM could be used routinely because it could detect carbapenemase activity with high sensitivity and specificity; however, the authors indicated the lack of determination of the carbapenemase enzyme type was a limitation of the study. Additionally, modification of dBLIM by adding specific beta-lactamase inhibitors such as phenyl boronic acid (PBA) and EDTA to identify class A and B carbapenemases or by adding an antibiotic combination containing cloxacillin and clavulanic acid (MEM or cefotaxime) to distinguish AmpC beta-lactamases from ESBLs has been suggested for further investigation by Bianco et al. (19). In this context, our findings revealed that the sensitivity and specificity of dBLIMplus in detecting carbapenemase activity in Enterobacterales isolates were similar to those of dBLIM and also that dBLIMplus could determine *bla*_KPC_, *bla*_NDM,_ and *bla*_OXA-48_ carbapenemases in Enterobacterales isolates with 39.3% sensitivity and 100% specificity in 6 h. Visual assessment of zone diameters became more apparent at 12 and 18 h. However, dBLIMplus demonstrated the highest sensitivity and specificity for determining carbapenemase types at 6 h. Therefore, when dBLIMplus is used to detect carbapenemase activity and determine the carbapenemase enzyme type in routine laboratory practice, a 6-h reading time is recommended for optimal interpretation. We evaluated the use of dBLIMplus only in aerobic BC bottles as Bianco et al. reported a significant decrease in sensitivity for detecting certain enzyme types (MBL) with both dBLIM and deBLIM in anaerobic BC bottles. One of the limitations of our study is that we did not evaluate the performance of dBLIMplus in anaerobic BC bottles.

In 2021, Bianco et al. described the direct EDTA-modified beta-lactam inactivation method (deBLIM), a variant of dBLIM that could detect serine carbapenemase or MBL-producing Enterobacterales directly from positive BC bottles in 6 h. The authors reported that the limitations of the study were the inability of deBLIM to distinguish subtypes of serine carbapenemases (e.g., KPC and OXA) and the inability to evaluate the usability of deBLIM among isolates containing more than one carbapenemase type (22). Unlike deBLIM, we demonstrated that dBLIMplus can distinguish subtypes of serine carbapenemases, and this suggests that dBLIMplus provides a wider interpretation potential. One *bla*_KPC_ isolate was not detected by dBLIMplus due to a borderline zone diameter difference (Z_C-A_ = 3 mm). The isolate was also not detected by CDT. This may be attributed to low-level enzyme expression, thus leading to inaccurate detection of activity in the phenotypic assay. In addition, the increase in Z_E_ from 10 mm at 6 h to 12 mm at 12 h observed in dBLIMplus for the three *bla*_OXA-48_ isolates resulted in decreased sensitivity of dBLIMplus at the 12- and 18-h time points compared to the 6-h time point. This finding may be explained by either reduced enzyme expression over time or decreased diffusion into the medium in *bla*_OXA-48_ isolates. However, it should not be ignored that isolates with indeterminate results in dBLIMplus may show resistance due to another carbapenemase enzyme or another mechanism that cannot be detected by the BD MAX Check-Points CPO test (23, 24). Furthermore, the fact that we could not perform whole-genome sequencing to reveal the presence of any resistance genes in the strains with indeterminate results is a limitation of our study. In various studies conducted to determine the carbapenemase enzymes, it has been reported that indeterminate results were often due to hypermucoid isolates (6, 25). In our study, none of the strains for which the dBLIMplus result was incompatible with PCR or for which an indeterminate result was obtained were hypermucoid.

Rapid antimicrobial susceptibility testing (RAST) defined by EUCAST and different protocols described by numerous studies have assessed and confirmed various approaches aimed at identifying carbapenemase activity directly from positive BC bottles; however, it was reported that carbapenemase enzyme types also could not be determined in these studies as in dBLIM (26, 27). On the other hand, some researchers have suggested that CIM could be modified to determine carbapenemase enzymes by adding beta-lactamase inhibitors such as cloxacillin, PBA, and sodium mercaptoacetate, and new methods have been developed and evaluated to determine the carbapenemase enzymes earlier. Although the sensitivity and specificity of these methods in detecting carbapenemase activity and determining carbapenemase enzymes were found to be high, they gave results in a longer time than other protocols applied in positive BC bottles due to the need for cultivated bacterial colonies (6, 25). Unlike other protocols defined for positive BC bottles, the ability of dBLIMplus to determine not only carbapenemase activity but also carbapenemase enzyme type in a relatively short time of 6 h supports our hypothesis that dBLIMplus will be more effective in the management of antibiotic treatment.

In our study, the sensitivity of CDT in detecting carbapenemase activity was 94%, and its sensitivity in determining carbapenemase enzyme type was 33.9%. On the other hand, the sensitivity of dBLIMplus was 100% in detecting carbapenemase activity and 39.3% in determining carbapenemase enzyme type. The sensitivity-specificity of CDT to determine carbapenemase enzymes was 55.5%–100% in *bla*_KPC_, 58.1%–98.5% in MBL, and 58.3%–98.1% in *bla*_OXA-48_, and the sensitivity-specificity of dBLIMplus to determine carbapenemase enzymes was 77.8%–100% in *bla*_KPC_, 22.6%–100% in MBL, and 79.2%–98.1% in *bla*_OXA-48_, respectively. dBLIMplus demonstrated a notably faster turnaround time compared to the conventional CDT. dBLIMplus showed superior sensitivity for identifying *bla*_KPC_ and *bla*_OXA-48_ carbapenemases, whereas CDT demonstrated higher sensitivity for determining MBL.

In 32 isolates harboring more than one carbapenemase enzyme (31 *bla*_NDM+OXA-48_ and 1 *bla*_KPC+OXA-48_), CDT identified most as MBL-producing, while dBLIMplus identified most as OXA-48-producing. This may be due to the fact that the application steps of dBLIMplus allow OXA-48 expression while reducing MBL expression. The components of the BC bottles used in dBLIMplus may have also suppressed MBL expression. Furthermore, in strains containing more than one carbapenemase enzyme, both enzymes may have masked each other’s effects. This may make it difficult to distinguish the dominant carbapenemase enzyme in the phenotype. Although phenotypic tests such as mCIM/eCIM, CDT, and dBLIMplus are useful for confirming carbapenemase activity, definitive enzyme typing should be based on molecular methods, and phenotypic results should be interpreted with caution due to inconsistencies and incomplete identification of isolates containing more than one carbapenemase type in phenotypic tests. Additionally, dBLIMplus is a disk diffusion-based method and requires precise measurement of zone diameter in millimeters for accurate detection. Observer variability can lead to measurement errors, so all reading time points for each isolate should be measured and evaluated by the same observer.

As reported in various studies, the carbapenemase enzymes of isolates harboring more than one carbapenemase enzyme may not be determined by phenotypic tests (25, 28). The sensitivity of dBLIMplus for determining the carbapenemase enzyme was found to be 77.8% in *bla*_KPC_, 22.6% in MBL, and 72.9% in *bla*_OXA-48_. Among strains with a single carbapenemase gene, the sensitivity of dBLIMplus for determining the carbapenemase type was 87.5% for *bla*_KPC_ and 93.5% for *bla*_OXA-48_. Since the number of isolates containing a single carbapenemase enzyme in our study was relatively low, the sensitivity, especially in MBL, remained low compared to that in other studies determining carbapenemases. Another reason for the higher sensitivity reported in other studies was that isolates harboring more than one carbapenemase enzyme were excluded from the analyses. However, unlike previous studies, the number of isolates containing more than one carbapenemase enzyme in our study was high (6, 22, 25). Similar to other phenotypic tests, it should be emphasized that isolates harboring more than one carbapenemase type also represent a limitation for dBLIMplus. In addition, the absence of clinical strains producing VIM, IMP, and only NDM prevented us from assessing the applicability of the test for these specific carbapenemase enzymes. Nevertheless, the method is capable of determining whether a strain exhibits carbapenemase activity. Therefore, further studies are required to assess the ability of dBLIMplus to determine carbapenemases other than those produced by the isolates in our study, including those produced by other members of Enterobacterales and nonfermentative gram-negative bacterial species.

It has been reported that different zone diameter criteria could be obtained with different commercial disk sets or manually prepared combination disks used for determining carbapenemase enzymes (23). Therefore, validation should be performed with routinely used disks before using a different disk set for dBLIMplus, and the evaluation criteria should be determined according to the disk set used. Failing to validate the method with the specific disk set in use may lead to misinterpretation of results and reduced diagnostic accuracy. Moreover, this highlights the need for standardization and disk-specific calibration of interpretive criteria to ensure the reliability of dBLIMplus results across different laboratories.

Given that dBLIMplus results can be read and interpreted within 6 h, the test can be performed on BC bottles at various time points throughout the working day in clinical laboratories. Applying dBLIMplus to BC bottles that yield negative results after 5 days of incubation may help reflect diverse clinical conditions encountered in daily practice, including interpatient variability. Another important point in daily practice is that validation should be performed with routinely used disks before using a different disk set for dBLIMplus, and the evaluation criteria should be determined according to the disk set used. In this study, dBLIMplus is presented as a validated method for Enterobacterales isolates. When a gram-negative rod is observed on Gram stain following a positive blood culture signal, species identification may be performed using platforms capable of direct identification from positive blood culture bottles (e.g., MALDI-TOF MS or rapid molecular panels). If an Enterobacterales isolate is identified, dBLIMplus can then be applied. While laboratories using MALDI-TOF MS and multiplex PCR-based systems can directly identify bacteria and carbapenemase types (*bla*_KPC_, *bla*_NDM_, *bla*_OXA-48_, etc.), these systems do not fully demonstrate whether the carbapenemase is actively expressed in that bacterium. If the carbapenemase type determined by PCR-based systems is not reflected in the bacterial phenotype, the isolate may exhibit minimum inhibitory concentration values that are within the susceptible range for carbapenems. Consequently, the use of dBLIMplus as a phenotypic test will be useful in this aspect.

To summarize, the study demonstrates that dBLIMplus offers an effective phenotypic approach for the detection of carbapenemase activity and determining carbapenemase enzymes directly from positive BC bottles. The cost of determining carbapenemase enzymes per strain using molecular methods such as the BD MAX Check-Points CPO assay is approximately $40.80, compared to $6.30 with dBLIMplus. With its cost-effectiveness, dBLIMplus offers a convenient solution for incorporation into the routine practices of clinical microbiology laboratories, where it may be utilized alongside other approaches to facilitate the prompt detection of carbapenemase activity from positive BC bottles.

## SUPPLEMENTAL MATERIAL

Supplemental tables and figures spectrum.01913-25-s0001.pdf Tables S1 and S2, and Figures S1 and S2.

ASM does not own the copyrights to Supplemental Material that may be linked to, or accessed through, an article. The authors have granted ASM a non-exclusive, world-wide license to publish the Supplemental Material files. Please contact the corresponding author directly for reuse.
