# Supplementary material for: An improved method for rapid detection and characterization of carbapenemase-producing Enterobacterales directly from positive blood cultures: dBLIMplus
Source: Microbiol Spectr. 2026 Apr 21;14(6):e01913-25. doi: 10.1128/spectrum.01913-25 (PMC13228065; doi:10.1128/spectrum.01913-25)
Supplement: Supplemental tables and figures — Tables S1 and S2, and Figures S1 and S2. [file spectrum.01913-25-s0001.pdf]

Table S1. Ranges of zone diameter (mm) and zone diameter differences (mm) measured at 6-12-18 hours with each combination disk of validation strains grouped according to PCR results

**Carbapenemase Type**	**Time**	**Zone diameter ranges**					**Range of zone diameter differences**		
		Z_A	Z_B	Z_C	Z_D	Z_E	Z_{B-A}	Z_{C-A}	Z_{D-A}
**non-CPE (n=15)**	**6**	12-14	12-14	12-13	12-14	10-13	-1-1	-1-1	0-1
	**12**	12-14	12-14	13-14	12-14	10-14	-1-2	-1-2	0-2
	**18**	12-14	12-14	13-14	13-15	10-14	-1-2	-1-2	0-2
***bla*KPC (n=15)**	**6**	6	6	11-12	6-10	10-12	0	5-7	0-4
	**12**	6	6	11-12	6-10	11-12	0	5-6	0-4
	**18**	6	6	11-13	6-10	11-12	0	5-7	0-4
**MBL (n=15)**	**6**	6	11-13	6-10	6-10	9-12	5-7	0-4	0-4
	**12**	6	11-13	6-10	6-10	9-12	5-7	0-4	0-4
	**18**	6	11-14	6-10	6-10	9-12	5-8	0-4	0-4
***bla*OXA-48 (n=15)**	**6**	6-11	6-11	6-11	6-11	9-11	0-4	0-3	-1-2
	**12**	6-11	6-11	6-11	6-11	10-11	0-4	0-4	-1-3
	**18**	6-11	6-11	6-11	6-11	10-11	0-4	0-3	-1-2

Z: Zone Diameter; A: Penem 10 µg; B: Penem 10 µg + MBL inhibitor; C: Penem 10 µg + KPC inhibitor; D: Penem 10 µg +AmpC inhibitor; E: Temocillin + MBL inhibitör; non-CPE: non-carbapenemase-producing Enterobacterales

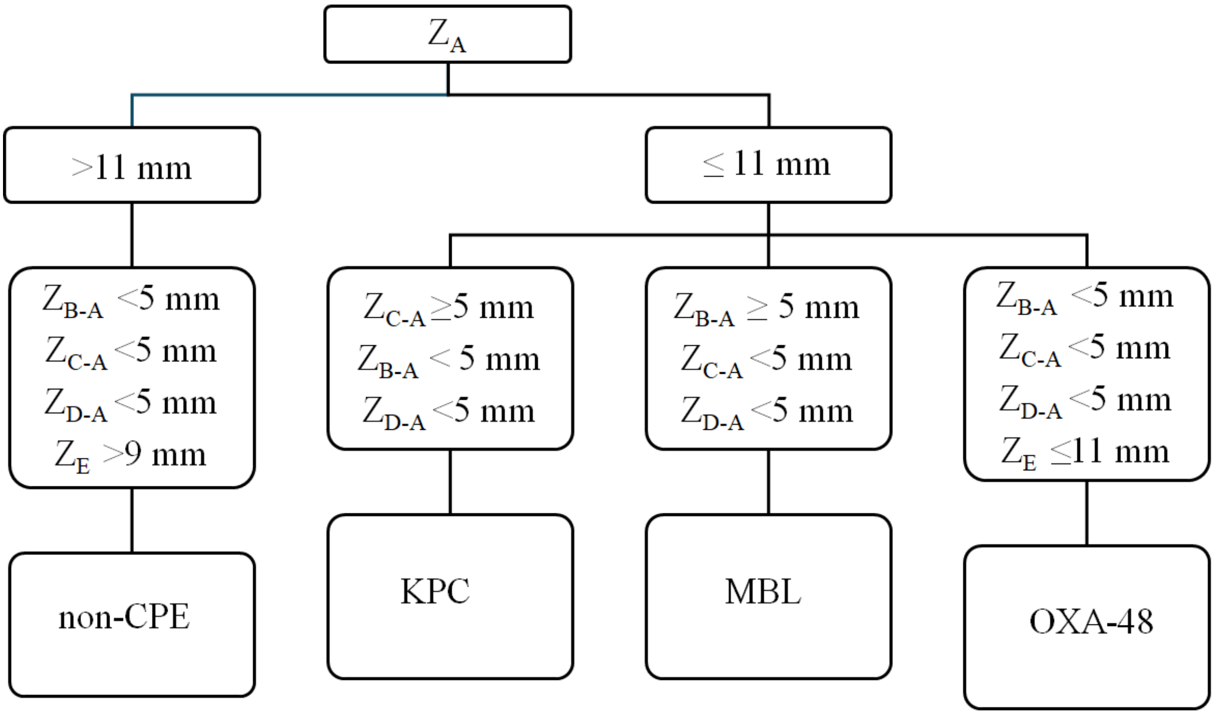

Z: Zone Diameter; A: Penem 10 µg; B: Penem 10 µg + MBL inhibitor; C: Penem 10 µg + KPC inhibitor; D: Penem 10 µg + AmpC inhibitor; E: Temocillin + MBL inhibitor

Figure S1. dBLIMplus evaluation criteria

Table S2. Ranges of zone diameter (mm) and zone diameter differences (mm) measured at 6-12-18 hours with each combination disk of clinical strains grouped according to PCR results

Carbapenemase Type	Time	Zone diameter ranges					Range of zone diameter differences		
		Z_A	Z_B	Z_C	Z_D	Z_E	Z_{B-A}	Z_{C-A}	Z_{D-A}
***bla*$_{KPC}$ (n=8)**	**6**	6-7	6	9-13	6-11	8-12	-1-0	**3**-7	0-4
	**12**	6-7	6	9-13	6-11	8-13	-1-0	**3**-7	0-4
	**18**	6-7	6	9-13	6-11	8-13	-1-0	**3**-7	0-4
***bla*$_{KPC+OXA-48}$ (n=1)**	**6**	6	6	9	8	10	0	3	2
	**12**	6	6	10	8	11	0	4	2
	**18**	6	6	10	8	11	0	4	2
***bla*$_{NDM+OXA-48}$ (n=31)**	**6**	6	6-13	6-10	6-12	8-12	0-7	0-4	0-**6**
	**12**	6	6-13	6-10	6-12	8-12	0-7	0-4	0-**6**
	**18**	6	6-13	6-10	6-12	8-12	0-7	0-4	0-**6**
***bla*$_{OXA-48}$ (n=16)**	**6**	6-11	6-12	6-12	6-12	9-12	0-2	0-4	0-3
	**12**	6-11	6-12	6-12	6-12	9-12	0-2	0-4	0-3
	**18**	6-11	6-12	6-12	6-13	9-12	0-4	0-4	0-3
**non-CPE (n=44)**	**6**	12-14	12-15	12-14	11-15	10-12	0-1	-1-0	0-1
	**12**	12-14	12-15	12-14	11-15	10-12	0-1	-1-0	0-1
	**18**	12-14	12-15	12-14	11-15	10-12	0-1	-1-1	0-1

Z: Zone Diameter; A: Penem 10 µg; B: Penem 10 µg + MBL inhibitor; C: Penem 10 µg + KPC inhibitor; D: Penem 10 µg +AmpC inhibitor; E: Temocillin + MBL inhibitör; non-CPE: non-carbapenemase-producing Enterobacterales

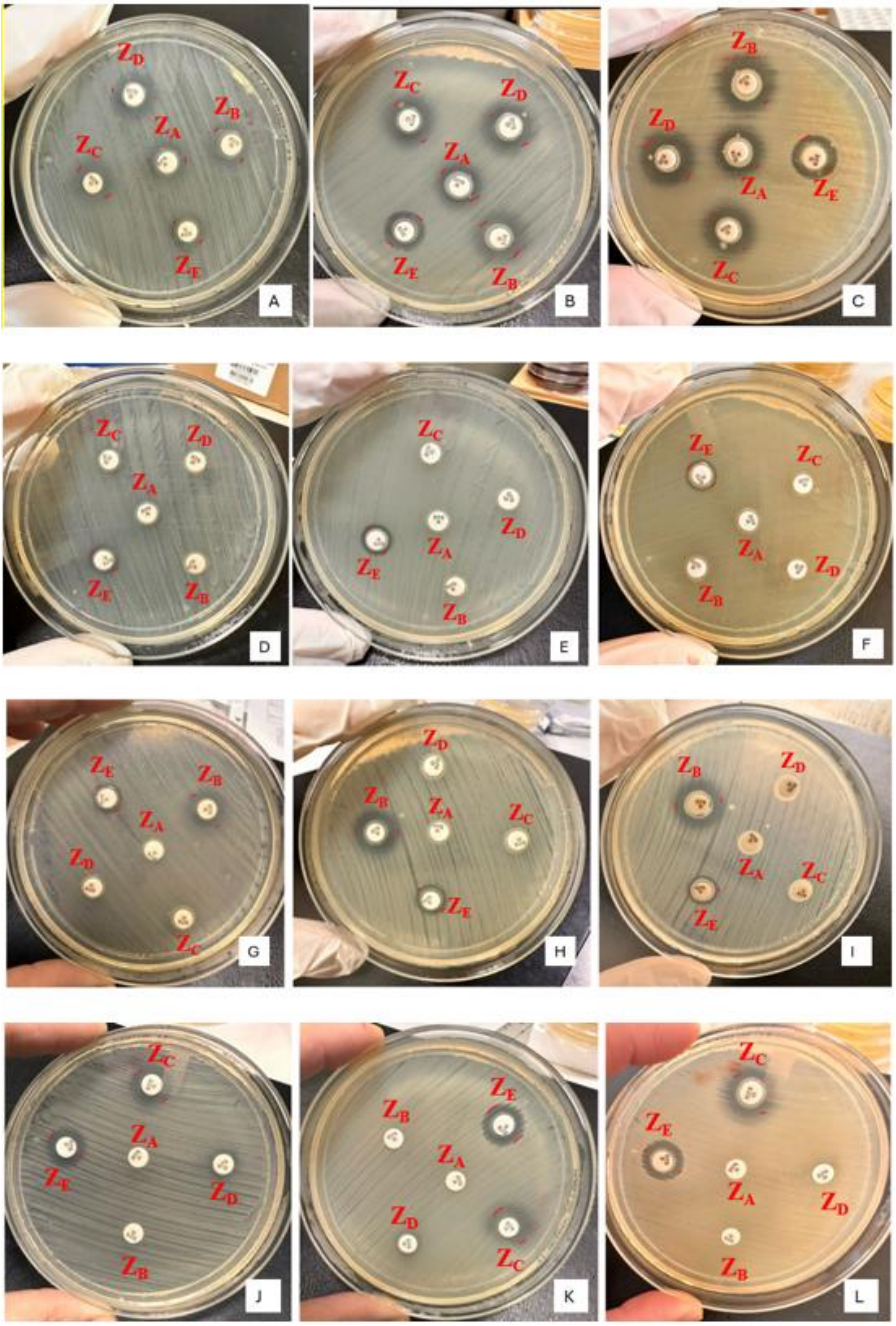

Figure S2. dBLIMplus inhibition zone diameters of carbapenemase negative strain (A-B-C), OXA-48 positive strain (D-E-F), MBL positive strain (G-H-I) and KPC positive strain (J-K-L) at 6, 12 and 18 hours, respectively
